# A case-crossover study of ST-elevation myocardial infarction and organic carbon and source-specific PM_2.5_ concentrations in Monroe County, New York

**DOI:** 10.3389/fpubh.2024.1369698

**Published:** 2024-08-01

**Authors:** Tianming Zhao, Philip K. Hopke, Mark J. Utell, Daniel P. Croft, Sally W. Thurston, Shao Lin, Frederick S. Ling, Yunle Chen, Catherine S. Yount, David Q. Rich

**Affiliations:** ^1^Department of Public Health Sciences, University of Rochester Medical Center, Rochester, NY, United States; ^2^Center for Air and Aquatic Resources Engineering and Sciences, Clarkson University, Potsdam, NY, United States; ^3^Division of Pulmonary and Critical Care, Department of Medicine, University of Rochester Medical Center, Rochester, NY, United States; ^4^Department of Environmental Medicine, University of Rochester Medical Center, Rochester, NY, United States; ^5^Department of Biostatistics and Computational Biology, University of Rochester Medical Center, Rochester, NY, United States; ^6^Department of Environmental Health, University at Albany School of Public Health, State University of New York, Rensselaer, NY, United States; ^7^Division of Cardiology, Department of Medicine, University of Rochester Medical Center, Rochester, NY, United States

**Keywords:** myocardial infarction, air pollution, PM_2.5_, source apportionment, organic carbon, case-crossover

## Abstract

**Background:**

Previous work reported increased rates of cardiovascular hospitalizations associated with increased source-specific PM_2.5_ concentrations in New York State, despite decreased PM_2.5_ concentrations. We also found increased rates of ST elevation myocardial infarction (STEMI) associated with short-term increases in concentrations of ultrafine particles and other traffic-related pollutants in the 2014–2016 period, but not during 2017–2019 in Rochester. Changes in PM_2.5_ composition and sources resulting from air quality policies (e.g., Tier 3 light-duty vehicles) may explain the differences. Thus, this study aimed to estimate whether rates of STEMI were associated with organic carbon and source-specific PM_2.5_ concentrations.

**Methods:**

Using STEMI patients treated at the University of Rochester Medical Center, compositional and source-apportioned PM_2.5_ concentrations measured in Rochester, a time-stratified case-crossover design, and conditional logistic regression models, we estimated the rate of STEMI associated with increases in mean primary organic carbon (POC), secondary organic carbon (SOC), and source-specific PM_2.5_ concentrations on lag days 0, 0–3, and 0–6 during 2014–2019.

**Results:**

The associations of an increased rate of STEMI with interquartile range (IQR) increases in spark-ignition emissions (GAS) and diesel (DIE) concentrations in the previous few days were not found from 2014 to 2019. However, IQR increases in GAS concentrations were associated with an increased rate of STEMI on the same day in the 2014–2016 period (Rate ratio [RR] = 1.69; 95% CI = 0.98, 2.94; 1.73 μg/m^3^). In addition, each IQR increase in mean SOC concentration in the previous 6 days was associated with an increased rate of STEMI, despite imprecision (RR = 1.14; 95% CI = 0.89, 1.45; 0.42 μg/m^3^).

**Conclusion:**

Increased SOC concentrations may be associated with increased rates of STEMI, while there seems to be a declining trend in adverse effects of GAS on triggering of STEMI. These changes could be attributed to changes in PM_2.5_ composition and sources following the Tier 3 vehicle introduction.

## Introduction

Ambient air pollution is a growing global public health concern (1), particularly fine particulate matter (<2.5 μm; PM_2.5_), which is the fifth-ranking global risk factor for mortality (2). Previous studies have demonstrated that elevations in PM_2.5_ concentrations increase the risk of cardiovascular events, partially attributable to the development of cardiometabolic risk factors (3–5). Further, some reported associations between increased ambient PM_2.5_ concentrations over the previous hours and days and the onset of myocardial infarctions (6–9). Our group and others have reported triggering of ST-elevation myocardial infarction (STEMI) by ambient pollutant concentrations (including PM_2.5_) in the previous few hours and days in some analyses (7, 8, 10–12), but not others (13, 14), suggesting work is needed to know what components or sources of PM may be driving any associations with STEMI.

PM_2.5_ is a complex mixture that includes organic compounds, elemental carbon, ions, and metal oxides, indicating PM_2.5_ components from various sources with different physicochemical characteristics and toxicological effects (15). Rich et al. observed larger odds of myocardial infarction associated with increased mass fractions of sulfate, nitrate, and ammonium and a lower elemental carbon mass fraction (16). Some reported an increased risk of cardiovascular admissions and mortality associated with short-term increases in elemental carbon and organic carbon concentrations (17–19), especially ischemic heart disease admissions (18). Our prior studies (13, 14) revealed increased STEMI rates associated with increases in ultrafine particles and black carbon concentrations in the previous hour. Thus, short-term changes in the concentration of specific PM components may trigger cardiovascular events including STEMI.

Organic carbon is classified as either primary organic carbon (POC) or secondary organic carbon (SOC), based on whether the constituent organic matter is generated from other compounds released into the atmosphere. POC is produced mostly from combustion processes, whereas SOC is formed through the oxidation of volatile organic compounds (VOCs) (20) and contains reactive oxygen species (10, 21, 22) that may induce oxidative stress and systemic inflammation, contributing to acute cardiovascular events (23, 24). To our knowledge, this work is the first epidemiological study to examine triggering of STEMI by short-term increases in SOC and POC concentrations.

PM_2.5_ sources emit particles with specific chemical characteristics that allow the identification and apportionment of PM to these sources. Previously, we employed source apportionment analyses to examine whether individual PM_2.5_ sources were associated with acute cardiovascular hospitalizations (25) and hospitalizations and emergency department (ED) visits for respiratory infections (26) and diseases (27) in adults across New York State from 2005 to 2016. Positive matrix factorization (PMF) was used to estimate the mass concentrations of particles corresponding to specific pollution sources at six urban sites (Buffalo, Rochester, Albany, Bronx, Manhattan, and Queens) in New York (28). There were 12 PM_2.5_ sources identified, including secondary sulfate (SS), secondary nitrate (SN), biomass burning (BB), diesel (DIE), spark-ignition emissions (GAS), pyrolyzed organic rich (OP), road dust (RD), aged sea salt (AGS), fresh sea salt (FSS), residual oil (RO), road salt (RS), and industrial (IND). Rich et al. reported that interquartile range (IQR) increases in GAS concentrations were associated with increased rates of hospitalization for cardiac arrhythmia and ischemic stroke on the same day, while IQR elevations in DIE concentrations were associated with elevated rates of hospitalization due to congestive heart failure and ischemic heart disease on the same day (25). Increased acute cardiovascular hospitalization rates were also associated with increased concentrations of RD, RO, and SN over the previous 1, 4, and 7 days.

Over the last decade, air quality in New York State has changed resulting from the reduction in sulfur concentrations in fuels, the closure of upwind coal-fired power plants, the energy transition from coal to natural gas, emissions controls on heavy-duty diesel vehicles, the Cross-State Air Pollution Rule, and the phase-out of residual oil for space heating in New York City. Furthermore, economic factors including the 2008 recession and the shift in the cost of natural gas relative to coal and oil, drove changes in electricity-generating unit technologies as well as air quality (29, 30). These changes resulted in substantial decreases in PM_2.5_ concentrations across New York State from 2005 to 2019 (29, 30), but compositional particle changes also occurred. We found that SS and SN concentrations decreased across New York State during 2005–2019, while GAS concentrations increased over this period (31, 32). We also observed decreased POC concentrations between 2005 and 2016, and an increase in SOC concentrations during 2014–2016 after a decline in the early years of the 2005–2013 period (28). In 2017, new regulations for Tier 3 light-duty vehicles began in New York State to improve emissions, and specifically to have lower SOC formation. These Tier 3 emission controls were only included in vehicles starting in 2017 and are not mandated for all vehicles until 2025. Any resulting reduction in emissions from these vehicles provided an opportunity to explore if the associations between the rates of STEMI and PM_2.5_ components and sources changed after their introduction in 2017 (i.e., early implementation period).

Using a dataset of patients whose STEMI was treated at the University of Rochester Medical Center (URMC) and ambient air pollutant concentrations from the monitoring station in Rochester from 2014 to 2019, we hypothesized that increases in concentrations of PM_2.5_ from motor vehicle and diesel sources (i.e., GAS and DIE) as well as SOC would be associated with an increased rate of STEMI. We also explored whether the introduction of Tier 3 light-duty vehicles from 2017 to 2019 (early Tier 3 implementation period) would lead to a reduced rate of STEMI associated with these specific PM_2.5_ sources/components, compared to the 2014–2016 period.

## Methods

**Study Population and Outcome Assessment**: This study included STEMI patients treated at the Cardiac Catheterization Laboratory (Cath Lab) at URMC in Rochester, New York from January 1, 2014 to December 31, 2019, who lived within 15 miles of the pollution monitoring site in Rochester. According to the American College of Cardiology (ACC)/American Heart Association (AHA) guidelines, a STEMI was defined as a myocardial infarction with an ST-segment elevation of >1 mm in two or more contiguous precordial leads, two or more adjacent limb leads, or a new or presumed new left bundle branch block in the presence of angina or angina equivalent on the presenting electrocardiogram (33). All STEMI events were diagnosed at the time of admission, with symptom onset date and time self-reported by each patient. If patients were unable to communicate, we obtained the information from their relatives. In terms of patients who experienced multiple STEMIs during the study period, if the subsequent STEMI event occurred ≥72 h after the previous one, it was counted as an additional event. In addition, demographic and clinical characteristics of patients were obtained from medical history and chart review. This study was approved by the University of Rochester Medical Center Research Subjects Review Board.

**Air Pollution and Meteorology Measurements**: PM_2.5_ compositional data was obtained from the U.S. Environmental Protection Agency (EPA) Chemical Speciation Network.^1^ Daily samples were collected and analyzed every third day in Rochester, and organic carbon, including primary organic carbon (POC) and secondary organic carbon (SOC), was measured using thermo-optical analysis. More details of the sampling methods, analytical protocols, and quality assurance and control were described previously (34). PM_2.5_ sources were identified using EPA positive matrix factorization (PMF) version 5, with further information on these analyses provided previously (28, 32). Eight PM_2.5_ sources identified in Rochester were used in this study, including secondary sulfate (SS), secondary nitrate (SN), spark-ignition emissions (GAS), diesel (DIE), road dust (RD), biomass burning (BB), pyrolyzed organic rich (OP), and road salt (RS). Daily ambient temperature and relative humidity in Rochester were measured at the Rochester International Airport and obtained from the National Weather Service (National Climate Data Center).^2^

**Study Design and Statistical Analyses**: We used a modified time-stratified case-crossover study design to estimate the rate of STEMI associated with each interquartile range increase in SOC, POC, and source-specific PM_2.5_ concentration in the previous 1, 3, and 6 days. For each STEMI, the standard time-stratified design (35, 36) would include the day of STEMI symptom onset as the case day (e.g., Wednesday July 12, 2023), and then use all of the same weekdays in the same calendar month (i.e., Wednesdays July 5, 19, and 26, 2023) as control days. All case and control days would be 7 days apart, and air pollutant concentrations (e.g., PM_2.5_ available for every day of the study period) would then be matched to each case and control day in the dataset for analysis. This time-stratified case-crossover design controls for non-time-varying potential confounders such as underlying medical conditions, long-term time trends, season, and weekday by design. Thus, we would not need to control for weekday in our conditional logistic regression models since each case and control day have the same value of weekday.

However, for our SOC, POC, and source-specific PM_2.5_ data (32) which were only available every 3rd day, there would be very few complete sets of case and control days for analysis using this standard time-stratified design (where case and referent days are 7 days apart). Therefore, we used a modified time-stratified design, where the day of STEMI symptom onset was again the case day (e.g., Wednesday July 12, 2023), but control days were now all the 6 days intervals before and after the case day within the same calendar month (e.g., Thursday July 6, 2023; Tuesday July 18, 2023; Monday July 24, 2023; Sunday July 30, 2023). Non-time varying potential confounders, such as underlying medical conditions, long-term time trends, and season, are still controlled by design. However, weekday is not, since case and referent days are no longer the same weekday. Further, air pollutant concentrations vary by weekday (28), and weekday has been included in acute health effect studies of air pollution and cardiorespiratory health events as a potential confounder (37–39). Therefore, using a conditional logistic regression model, stratified by each case–control set, we regressed case–control status (i.e., case = 1; control = 0) against the mean SOC concentration on lag day 0, adjusting for the mean residual PM_2.5_ concentration (i.e., residual PM_2.5_ = PM_2.5_–SOC; to control for potential confounding by non-SOC PM_2.5_), weekday, holidays, temperature (natural spline with 4 degrees of freed(39)om [df]), and relative humidity (linear term) on the same case and control days. We also separately re-ran this set of models for the mean SOC concentration on lag days 0–3 and 0–6. We then re-ran this set of model analyses for POC (including residual PM_2.5_ = PM_2.5_–POC) and each PM_2.5_ source in the same manner (residual PM_2.5_ = PM_2.5_–source-specific PM_2.5_ concentration [e.g., PM_2.5_–GAS]). For each model, we estimated the rate of STEMI associated with each interquartile range (IQR) increase in the specific pollutant concentration, and its 95% confidence interval (CI).

Next, we examined whether the rates of STEMI associated with each IQR increase in POC, SOC, and each PM_2.5_ source concentration differed between the 2014–2016 and 2017–2019 periods by adding an interaction term (e.g., SOC * 2017–2019_Period) to the model. Since we examined three lag times for each pollutant, statistical significance was defined as *p* < 0.017 (0.05/3). All data management and analyses were performed using SAS version 9.4 (^©^SAS Institute Inc., Cary, NC) and R version 4.2.3.

## Results

The demographic characteristics of the 186 patients with 188 STEMI events during the study period are provided in Table 1. The majority of these subjects were male (72.3%), white (83.4%), and non-Hispanic (94.7%) with a mean age of 62.8 years (standard deviation [SD]: 10.8 years). Compared to the 2014–2016 period, STEMI patients in the 2017–2019 period were older (64.4 ± 13.0 vs. 60.6 ± 10.8 years) and more likely to be smokers (45.0% vs. 35.5%). They were also less likely to be male (69.6% vs. 76.3%). More subjects had Medicare (28.2% vs. 4.3%) and Medicaid (10.9% vs. 4.3%) in the 2017–2019 period than those in the 2014–2016 period. Furthermore, a prior history of myocardial infarction and hypertension was more prevalent, and diabetes and dyslipidemia were less common among the participants in the 2017–2019 period relative to the 2014–2016 period. Participants in the 2017–2019 period stayed in the hospital for 4.2 days on average (SD = 4.3 days), shorter than the 2014–2016 period (Mean ± SD: 5.2 ± 12.5 days).

**Table 1:** Characteristics of STEMI patients by period.

Characteristics	2014–2016 (*N* = 76)^a^ n (%)	2017–2019 (*N* = 122)^a^ n (%)
**Age** (years)		
< 50	12 (15.8)	14 (12.5)
50–59	26 (34.2)	30 (26.8)
60–69	25 (32.9)	36 (32.1)
70–79	10 (13.2)	15 (13.4)
≥ 80	3 (3.9)	17 (15.2)
Mean ± SD	60.6 ± 10.8	64.4 ± 13.0
**Sex**		
Female	18 (23.7)	34 (30.4)
Male	58 (76.3)	78 (69.6)
**Race**		
Missing	0	1
Caucasian	63 (82.9)	93 (83.8)
African American	10 (13.2)	13 (11.7)
Asian	2 (2.6)	5 (4.5)
Others	1 (1.3)	0 (0)
**Ethnicity**		
Missing	1	0
Non-Hispanic	71 (94.7)	108 (96.4)
Hispanic/Latino	4 (5.3)	4 (3.6)
**Body Mass Index** (kg/m^2^)		
Normal (<25)	15 (19.7)	31 (27.7)
Overweight (25 ≤ ~ <30)	36 (47.4)	40 (35.7)
Obesity (30 ≤ ~ <35)	15 (19.7)	32 (28.6)
Severe Obesity (≥35)	10 (13.2)	9 (8.0)
Mean ± SD	28.8 ± 4.9	28.4 ± 5.6
**Smoking**		
Missing	0	12
Yes	27 (35.5)	45 (45.0)
No	49 (64.5)	55 (55.0)
**Health Insurance**		
Missing	6	2
Private	60 (85.7)	67 (60.9)
Medicare	3 (4.3)	31 (28.2)
Medicaid	3 (4.3)	12 (10.9)
No insurance	3 (4.3)	0 (0)
Other (military, non-US)	1 (1.4)	0 (0)
**Clinical Presentation**		
Prior Myocardial Infarction	7 (9.2)	20 (17.9)
Prior Percutaneous Coronary Intervention	7 (9.2)	14 (12.5)
Prior Coronary Artery Bypass Graft	4 (5.3)	3 (2.7)
Cardiovascular Disease	5 (6.6)	9 (8.0)
Hypertension^b^	53 (69.7)	75 (84.3)
Dyslipidemia	42 (55.3)	55 (49.1)
Diabetes	21 (27.6)	25 (22.3)
Prior Heart Failure	1 (1.3)	4 (3.6)
Family History Coronary Artery Disease	16 (21.1)	20 (17.9)
Prior Peripheral Arterial Disease^b^	3 (4.0)	3 (2.7)
Current Dialysis	1 (1.3)	0 (0)
Chronic Lung Disease	8 (10.5)	9 (8.0)
Length of Stay (days)^c^ Mean ± SD	5.2 ± 12.5	4.2 ± 4.3

For any given characteristic, the denominator of percentage is the number of STEMIs events with available data on that characteristic. SD, Standard Deviation.

a Ns were the number of STEMI events. There was a total of 188 STEMI events among 186 patients.

b The variable of hypertension had 23 missing values in the period of 2017–2019. The variable of prior peripheral arterial disease had one missing value in the 2014–2016 period.

c One outlier (length of stay = 347 days) removed from the period of 2014–2016.

Daily concentrations of POC, SOC, and PM_2.5_ sources are summarized in Table 2. From the 2014–2016 period to the 2017–2019 period, the median concentration of POC and SOC increased by 34.4% (2014–2016: 0.32 μg/m^3^; 2017–2019: 0.43 μg/m^3^) and 41.8% (2014–2016: 0.55 μg/m^3^; 2017–2019: 0.78 μg/m^3^), respectively. In terms of PM_2.5_ sources, there were substantial increases in median concentrations of SN (100%; 2014–2016: 0.15 μg/m^3^; 2017–2019: 0.30 μg/m^3^), GAS (125%; 2014–2016: 0.96 μg/m^3^; 2017–2019: 2.16 μg/m^3^), and OP (71.4%; 2014–2016: 0.21 μg/m^3^; 2017–2019: 0.36 μg/m^3^). There were large decreases in median concentrations of SS (48%; 2014–2016: 1.13 μg/m^3^; 2017–2019: 0.59 μg/m^3^) and BB (59%; 2014–2016: 0.44 μg/m^3^; 2017–2019: 0.18 μg/m^3^), but little to no change in PM_2.5_, DIE, RD, and RS concentrations. SOC was moderately correlated with PM_2.5_ (*r* = 0.53) and POC (*r* = 0.51; Table 3), while SS was moderately correlated with PM_2.5_ (*r* = 0.64) and OP (*r* = 0.58). SN was negatively correlated with temperature (*r* = −0.55). DIE was positively correlated with RD (*r* = 0.14) and OP (*r* = 0.03), but negatively correlated with other PM_2.5_ sources (SS: *r* = −0.14; SN: *r* = −0.06; GAS: *r* = −0.12; BB: *r* = −0.23; RS: −0.14), although these correlations were weak.

**Table 2:** Distribution of daily concentrations (μg/m^3^) of organic carbon and PM_2.5_ sources and weather characteristics by period^a^.

	N	Mean	Min	P5	P25	Median	P75	P95	Max	IQR
**PM**_**2.5**_ – **From composition measurements**										
All years	716	6.90	−0.73	1.81	3.48	5.72	7.95	12.65	88.11	4.47
2014–2016	288	8.02	−0.73	1.64	3.51	5.69	8.78	13.27	88.11	5.27
2017–2019	428	6.13	0.18	1.88	3.48	5.74	7.60	12.49	21.73	4.13
**Primary Organic Carbon (POC)**										
All years	716	0.56	−0.47	0.00	0.19	0.37	0.70	1.72	3.74	0.51
2014–2016	288	0.47	0.00	0.00	0.17	0.32	0.54	1.33	3.74	0.37
2017–2019	428	0.63	−0.47	0.00	0.23	0.43	0.79	2.13	3.57	0.55
**Secondary Organic Carbon (SOC)**										
All years	716	0.83	0.00	0.10	0.42	0.71	1.06	1.61	11.63	0.65
2014–2016	288	0.83	0.00	0.00	0.30	0.55	1.06	1.74	11.63	0.76
2017–2019	428	0.83	0.13	0.21	0.51	0.78	1.06	1.56	2.62	0.56
**PM**_**2.5**_ – **From source measurements**										
All years	716	6.5	0.9	2.2	3.8	6.0	8.2	12.8	21.7	4.4
2014–2016	288	6.7	1.0	2.1	3.8	6.0	8.5	13.5	20.1	4.7
2017–2019	428	6.4	0.9	2.2	3.8	6.0	7.7	12.5	21.7	3.9
**Secondary Sulfate (SS)**										
All years	716	1.33	−1.29	−0.40	0.15	0.78	2.06	4.91	11.48	1.90
2014–2016	288	1.92	−0.75	−0.25	0.17	1.13	3.12	6.29	11.48	2.96
2017–2019	428	0.99	−1.29	−0.54	0.11	0.59	1.41	4.20	5.72	1.30
**Secondary Nitrate (SN)**										
All years	716	0.89	−0.55	−0.19	0.01	0.26	1.14	4.19	10.55	1.12
2014–2016	288	0.67	−0.40	−0.29	−0.03	0.15	0.98	2.90	7.92	1.01
2017–2019	428	1.02	−0.55	−0.17	0.02	0.30	1.19	4.73	10.55	1.17
**Spark-ignition Emissions (GAS)**										
All years	716	1.99	−0.16	−0.03	0.96	1.79	2.68	4.60	8.05	1.73
2014–2016	288	1.28	−0.16	−0.06	0.42	0.96	1.91	4.01	5.07	1.48
2017–2019	428	2.41	−0.10	0.77	1.42	2.16	2.99	5.00	8.05	1.56
**Diesel (DIE)**										
All years	716	0.61	−0.29	0.05	0.34	0.60	0.81	1.19	2.43	0.47
2014–2016	288	0.61	−0.12	0.09	0.33	0.60	0.79	1.25	2.10	0.46
2017–2019	428	0.61	−0.29	0.02	0.35	0.60	0.83	1.19	2.43	0.49
**Road Dust (RD)**										
All years	716	0.21	−0.12	−0.02	0.07	0.16	0.32	0.61	1.65	0.25
2014–2016	288	0.24	−0.07	0.01	0.09	0.18	0.34	0.61	1.65	0.25
2017–2019	428	0.20	−0.12	−0.04	0.06	0.15	0.30	0.54	1.18	0.24
**Biomass Burning (BB)**										
All years	716	0.42	−0.24	−0.09	0.00	0.25	0.63	1.47	3.23	0.63
2014–2016	288	0.58	−0.24	−0.02	0.16	0.44	0.78	1.90	3.23	0.62
2017–2019	428	0.32	−0.23	−0.12	−0.05	0.18	0.53	1.25	2.09	0.58
**Road Salt (RS)**										
All years	716	0.07	−0.06	−0.02	0.00	0.01	0.03	0.36	1.89	0.03
2014–2016	288	0.10	−0.03	−0.01	0.00	0.01	0.04	0.56	1.89	0.04
2017–2019	428	0.05	−0.06	−0.02	0.00	0.01	0.03	0.25	1.62	0.03
**Pyrolyzed Organic Rich (OP)**										
All years	716	0.38	−0.21	−0.02	0.14	0.33	0.55	0.99	2.47	0.41
2014–2016	288	0.33	−0.12	−0.02	0.08	0.21	0.53	1.11	1.59	0.45
2017–2019	428	0.41	−0.21	−0.02	0.22	0.36	0.56	0.95	2.47	0.34
**Temperature** (°C)										
All years	716	12.47	−13.97	−5.61	4.55	13.93	20.67	26.03	29.46	−1.65
2014–2016	288	13.81	−11.64	−6.01	7.49	16.00	21.24	27.83	28.83	−4.03
2017–2019	428	11.55	−13.97	−5.00	2.74	12.69	20.42	25.49	29.46	−0.10
**Relative Humidity** (%)										
All years	716	67.02	30.33	45.46	58.08	66.33	75.85	89.56	98.25	17.77
2014–2016	288	62.97	30.33	42.98	54.38	61.75	71.17	86.37	96.00	16.79
2017–2019	428	69.78	31.26	50.63	60.25	71.29	78.46	90.37	98.25	18.21

Min, Minimum; P5, 5th Percentile; P25, 25th Percentile; P75, 75th Percentile; P95, 95th Percentile; Max, Maximum; IQR, Interquartile Range.

a Data on control periods and lag day 0 were used.

**Table 3:** Pearson correlation coefficients between daily pollutant concentrations (μg/m^3^) and weather measurements during 2014–2019^a^.

Compositional PM_2.5_ Measurements											
	**PM**_**2.5**_	**POC**	**SOC**	**Temp**	**RH**						
**PM**_**2.5**_	1.00										
**POC**	0.34	1.00									
**SOC**	0.53	0.51	1.00								
**Temp**	0.19	0.47	0.24	1.00							
**RH**	−0.06	−0.11	−0.09	−0.13	1.00						
Source-specific PM_2.5_ Measurements											
	**PM**_**2.5**_	**SS**	**SN**	**GAS**	**DIE**	**RD**	**BB**	**RS**	**OP**	**Temp**	**RH**
**PM**_**2.5**_	1.00										
**SS**	0.64	1.00									
**SN**	0.39	−0.08	1.00								
**GAS**	0.48	0.16	−0.12	1.00							
**DIE**	−0.14	−0.14	−0.06	−0.12	1.00						
**RD**	0.15	0.23	−0.03	−0.06	0.14	1.00					
**BB**	0.45	0.32	0.33	0.03	−0.23	0.16	1.00				
**RS**	0.15	0.00	0.34	−0.13	−0.14	0.04	0.22	1.00			
**OP**	0.45	0.58	−0.15	0.31	0.03	0.13	−0.01	−0.15	1.00		
**Temp**	0.22	0.39	−0.55	0.40	0.15	0.11	−0.19	−0.32	0.44	1.00	
**RH**	−0.02	−0.11	0.13	−0.08	0.11	−0.27	−0.08	0.01	−0.04	−0.13	1.00

POC, primary organic carbon; SOC, secondary organic carbon; SS, secondary sulfate; SN, secondary nitrate; GAS, spark-ignition emissions; DIE, diesel; RD, road dust; BB, biomass burning; RS, road salt; OP, pyrolyzed organic rich; Temp, temperature (°C); RH, relative humidity (%).

a Data on control periods and lag day 0 were used.

Inconsistent with our *a priori* hypothesis, interquartile range (IQR) increases in mean SOC concentrations on the same day, and previous 3 and 6 days, were not associated with increased rates of STEMI (Table 4). However, each IQR increase in mean SOC concentration in the previous 6 days was associated with an imprecise, but suggestive 14% increased rate of STEMI (Rate ratio [RR] = 1.14; 95% CI = 0.89, 1.45). Similarly, inconsistent with our *a priori* hypotheses, IQR increases in GAS and DIE on the same day, and previous 3 and 6 days, were not associated with increased rates of STEMI. There were no increased rates of STEMI associated with POC or any other source specific PM_2.5_ concentration at any lag time (Table 4).

**Table 4:** Rates of STEMI associated with each interquartile range increase in concentrations (μg/m^3^) of organic carbon and PM_2.5_ sources in a multivariable model^a^.

Lag day	IQR^b^	N of STEMI	OR	95% CI	*p*-value
**Primary Organic Carbon (POC)**					
0	0.51	180	0.94	0.77, 1.15	0.565
0–3	0.53	177	0.89	0.63, 1.26	0.527
0–6	0.55	182	0.87	0.57, 1.34	0.527
**Secondary Organic Carbon (SOC)**					
0	0.65	180	1.00	0.88, 1.14	0.950
0–3	0.45	177	1.06	0.84, 1.33	0.640
0–6	0.42	182	1.14	0.89, 1.45	0.306
**Secondary Sulfate (SS)**					
0	1.90	170	1.00	0.77, 1.31	0.972
0–3	1.51	146	1.18	0.88, 1.59	0.226
0–6	1.61	161	0.99	0.70, 1.42	0.969
**Secondary Nitrate (SN)**					
0	1.12	170	0.95	0.79, 1.13	0.530
0–3	1.18	146	0.90	0.68, 1.19	0.467
0–6	1.24	161	0.93	0.64, 1.34	0.687
**Spark-ignition Emissions (GAS)**					
0	1.73	170	0.97	0.72, 1.31	0.828
0–3	1.52	146	0.92	0.62, 1.36	0.681
0–6	1.33	161	0.96	0.63, 1.46	0.849
**Diesel (DIE)**					
0	0.47	170	0.86	0.66, 1.12	0.254
0–3	0.36	146	0.89	0.63, 1.25	0.492
0–6	0.34	161	1.06	0.72, 1.56	0.765
**Road Dust (RD)**					
0	0.25	170	0.99	0.77, 1.28	0.941
0–3	0.18	146	0.96	0.71, 1.31	0.809
0–6	0.18	161	1.01	0.72, 1.42	0.944
**Biomass Burning (BB)**					
0	0.63	170	0.88	0.67, 1.15	0.335
0–3	0.47	146	1.00	0.72, 1.39	0.977
0–6	0.46	161	0.76	0.54, 1.06	0.104
**Road Salt (RS)**					
0	0.03	170	0.99	0.95, 1.02	0.418
0–3	0.04	146	0.98	0.90, 1.07	0.687
0–6	0.04	161	1.00	0.93, 1.09	0.931
**Pyrolyzed Organic Rich (OP)**					
0	0.41	170	1.03	0.79, 1.34	0.833
0–3	0.34	146	0.99	0.69, 1.43	0.972
0–6	0.31	161	1.08	0.73, 1.61	0.687

STEMI, ST-elevation myocardial infarction; IQR, interquartile range; OR, odds ratio; CI, confidence interval.

a For organic carbons, adjustments included the residuals between PM_2.5_ (from composition measurements) and POC or SOC for the corresponding lag day, weekday, holidays, and temperature (a natural spline with 4 degrees of freedom) and relative humidity (a linear term) for the corresponding lag day. For PM2.5 sources, adjustments included the residuals between PM_2.5_ (from source measurements) and specific PM_2.5_ source for the corresponding lag day, weekday, holidays, and temperature (a natural spline with 4 degrees of freedom) and relative humidity (a linear term) for the corresponding lag day.

b The IQR for the corresponding pollutant and lag day was calculated using data from the control periods during all years.

We also explored whether rates of STEMI were separately associated with increased SOC, POC, and source-specific PM_2.5_ concentrations in 2014–2016 and 2017–2019 and whether these period-specific rate ratios were different (Table 5). Although increased SOC, POC, and source-specific PM_2.5_ concentrations were not associated with increased rates of STEMI during either period at any lag time, each IQR increase in SS on lag day 0 was associated with a decreased rate of STEMI (RR = 0.63; 95% CI = 0.40, 0.98) during 2017–2019. Further, although imprecise, rate ratios were substantially greater than 1.0 for POC, SOC, SS, and GAS for most lag times in 2014–2016.

**Table 5:** Rates of STEMI associated with each interquartile range in concentrations (μg/m^3^) of organic carbon and PM_2.5_ sources by period, from a model including the interaction between pollutant concentrations and period^a^.

Lag day	IQR^b^	2014–2016				2017–2019				Period Interaction *p*-value
		N of STEMI	OR	95% CI	*p*-value	N of STEMI	OR	95% CI	*p*-value	
**Primary Organic Carbon (POC)**										
0	0.51	74	1.20	0.90, 1.60	0.219	106	0.79	0.60, 1.03	0.083	0.023
0–3	0.53	72	1.48	0.79, 2.78	0.226	105	0.78	0.52, 1.15	0.202	0.066
0–6	0.55	74	1.79	0.83, 3.86	0.139	108	0.69	0.43, 1.12	0.130	0.028
**Secondary Organic Carbon (SOC)**										
0	0.65	74	1.02	0.90, 1.15	0.769	106	0.83	0.57, 1.21	0.335	0.294
0–3	0.45	72	1.13	0.88, 1.44	0.350	105	0.90	0.62, 1.30	0.569	0.274
0–6	0.42	74	1.27	0.96, 1.67	0.092	108	0.84	0.54, 1.30	0.431	0.103
**Secondary Sulfate (SS)**										
0	1.90	67	1.22	0.90, 1.65	0.203	103	0.63	0.40, 0.98	0.040	**0.006**
0–3	1.51	50	1.30	0.93, 1.80	0.120	96	0.90	0.55, 1.48	0.675	0.170
0–6	1.61	59	1.16	0.80, 1.69	0.426	102	0.60	0.33, 1.09	0.093	0.039
**Secondary Nitrate (SN)**										
0	1.12	67	0.60	0.38, 0.97	0.037	103	1.01	0.85, 1.21	0.908	0.039
0–3	1.18	50	0.73	0.35, 1.53	0.406	96	0.93	0.69, 1.24	0.616	0.546
0–6	1.24	59	0.47	0.19, 1.11	0.086	102	1.10	0.74, 1.64	0.649	0.071
**Spark-ignition Emissions (GAS)**										
0	1.73	67	1.69	0.98, 2.94	0.061	103	0.79	0.55, 1.13	0.199	**0.016**
0–3	1.52	50	1.42	0.74, 2.72	0.295	96	0.75	0.47, 1.21	0.240	0.109
0–6	1.33	59	1.63	0.87, 3.04	0.125	102	0.70	0.42, 1.17	0.173	0.028
**Diesel (DIE)**										
0	0.47	67	0.67	0.41, 1.07	0.096	103	0.97	0.70, 1.33	0.834	0.196
0–3	0.36	50	0.75	0.42, 1.34	0.326	96	0.97	0.64, 1.47	0.884	0.471
0–6	0.34	59	0.93	0.52, 1.66	0.809	102	1.16	0.71, 1.88	0.551	0.554
**Road Dust (RD)**										
0	0.25	67	0.91	0.63, 1.32	0.635	103	1.07	0.75, 1.51	0.718	0.539
0–3	0.18	50	0.84	0.52, 1.35	0.464	96	1.06	0.72, 1.55	0.775	0.428
0–6	0.18	59	0.77	0.47, 1.25	0.291	102	1.33	0.84, 2.11	0.227	0.097
**Biomass Burning (BB)**										
0	0.63	67	1.05	0.74, 1.48	0.797	103	0.70	0.47, 1.05	0.084	0.125
0–3	0.47	50	1.15	0.74, 1.79	0.522	96	0.86	0.54, 1.37	0.537	0.352
0–6	0.46	59	0.83	0.54, 1.28	0.405	102	0.66	0.40, 1.11	0.118	0.500
**Road Salt (RS)**										
0	0.03	67	0.98	0.94, 1.03	0.490	103	0.99	0.94, 1.04	0.663	0.882
0–3	0.04	50	1.01	0.90, 1.14	0.811	96	0.95	0.84, 1.08	0.413	0.443
0–6	0.04	59	0.99	0.90, 1.09	0.846	102	1.03	0.90, 1.19	0.649	0.627
**Pyrolyzed Organic Rich (OP)**										
0	0.41	67	1.33	0.91, 1.94	0.143	103	0.86	0.61, 1.21	0.395	0.070
0–3	0.34	50	1.32	0.70, 2.50	0.396	96	0.91	0.61, 1.36	0.653	0.289
0–6	0.31	59	1.49	0.83, 2.70	0.184	102	0.92	0.58, 1.45	0.707	0.150

STEMI, ST-elevation myocardial infarction; IQR, interquartile range; OR, odds ratio; CI, confidence interval.

a For organic carbons, adjustments included the residuals between PM_2.5_ (from composition measurements) and POC or SOC for the corresponding lag day, weekday, holidays, and temperature (a natural spline with 4 degrees of freedom) and relative humidity (a linear term) for the corresponding lag day. For PM_2.5_ sources, adjustments included the residuals between PM_2.5_ (from source measurements) and specific PM_2.5_ source for the corresponding lag day, weekday, holidays, and temperature (a natural spline with 4 degrees of freedom) and relative humidity (a linear term) for the corresponding lag day.

b The IQR for the corresponding pollutant and lag day was calculated using data from the control periods during all years.

Next, there were significant differences (*p* < 0.017) in period specific rate ratios for SS on lag day 0 (2014–2016: RR = 1.22, 95% CI = 0.90, 1.65; 2017–2019: RR = 0.63, 95% CI = 0.40, 0.98) and GAS on lag day 0 (2014–2016: RR = 1.69, 95% CI = 0.98, 2.94; 2017–2019: RR = 0.79, 95% CI = 0.55, 1.13; Table 5). Although not statistically significant, there were substantial differences in rate ratios for POC on lag day 0 (2014–2016: RR = 1.20, 95% CI = 0.90, 1.60; 2017–2019: RR = 0.79, 95% CI = 0.60, 1.03) and lag day 6 (2014–2016: RR = 1.79, 95% CI = 0.83, 3.86; 2017–2019: RR = 0.69, 95% CI = 0.43, 1.12), and GAS on lag day 6 (2014–2016: RR = 1.63, 95% CI = 0.87, 3.04; 2017–2019: RR = 0.70, 95% CI = 0.42, 1.17).

## Discussion

Inconsistent with our *a priori* hypothesis, we did not observe an increase in rates of STEMI associated with increased PM_2.5_ concentrations from GAS and DIE sources (i.e., markers of traffic pollution) in Rochester, New York, from 2014 to 2019. However, an increase in GAS concentrations was associated with the increased rate of STEMI on the same day in the 2014–2016 period, but not in the 2017–2019 period. In addition, each IQR increase in mean SOC concentration in the previous 6 days was associated with an increased rate of STEMI in 2014–2016, despite the lack of precision. Similarly, we generally did not find increased rates of STEMI associated with increased POC or any other source-specific PM_2.5_ concentrations. In the exploratory analysis by period, we found a negative association between SS concentration on the same day and the rate of STEMI during 2017–2019 (i.e., after Tier 3 vehicle introduction). In addition, even in this early Tier 3 implementation period (2017–2019), there were significant differences in the rates of STEMI associated with SS and GAS on lag day 0 between the 2014–2016 and 2017–2019 periods. Further work will be needed to examine full implementation of Tier 3 through 2025.

This finding of a decreased rate of STEMI associated with increased SS concentrations in 2017–2019 may be spurious, and just a result of an examining effect modification of an overall null association in secondary analyses. In this case, the overall effect across the 2014–2019 period is null (i.e., RR = 1.0) and we find an increased rate ratio in 2014–2016, so there must be a decreased rate ratio in 2017–2019. Two prior studies, also using STEMI data from the Cath Lab, air pollution data from the Rochester monitoring station, and the same analysis methods, found no associations between the rate of STEMI and PM_2.5_ concentration in the previous few hours and days in Rochester during the 2005–2016 (13) and 2014–2019 periods (14). Our null findings with PM_2.5_ sources and components over 2014–2019 are consistent with these analyses.

However, there were also studies suggesting positive associations using earlier data from the same sources and the same analysis methods (7, 8). After rescaling effects with the same IQR (4.47 μg/m^3^) for PM_2.5_ used in our analysis, Evans et al. reported a 10% increase in the rate of STEMI associated with each 4.47 μg/m^3^ increase in PM_2.5_ concentration in the previous hour (RR = 1.10, 95% CI = 0.99, 1.23) during 2007–2012 (8). A similar result (IQR = 4.47 μg/m^3^, RR = 1.11, 95% CI = 1.01, 1.22) was also found by Gardner et al. using data from 2007 to 2010 (7). Some, but not all, other case-crossover studies also reported that increased PM_2.5_ concentrations were associated with an increased risk of STEMI. For example, each 4.47 μg/m^3^ increase in PM_2.5_ concentration was associated with estimated RRs of 1.06 (95% CI = 1.01, 1.12) in Utah, United States, between 1993 and 2014 (10), 1.02 (95% CI = 1.00, 1.05) in Beijing in 2014 (12), and 1.72 (95% CI = 1.00, 2.19) in Suzhou, China, from 2013 to 2016 (11). The discrepancy in these results is likely due to different study locations and changes in PM composition over time. However, it could also be due to differences in STEMI patient characteristics over time and differences in lag patterns of associations.

**Figure 1:**
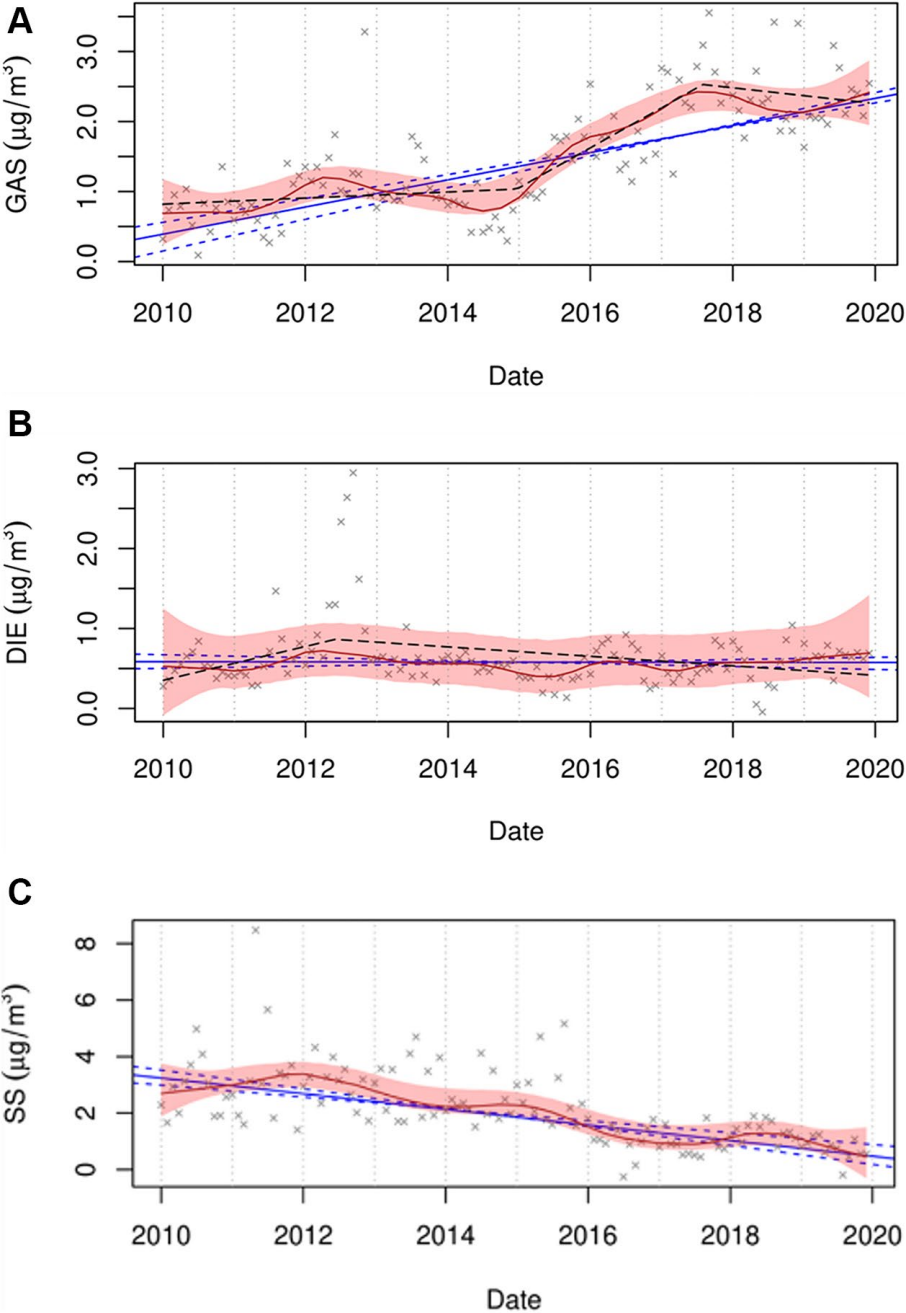
Trend plots for GAS **(A)**, DIE **(B)**, and SS **(C)** for the period of 2010 to 2019. The red line represents the seasonal by loess trends (STL), with 95% confidence bound as a shaded area. The blue line represents the Thiel-Sen monotonic trend, with a 95% confidence interval as dashed lines. The black dashed line represents the piecewise trend. GAS, DIE, and SS represent spark-ignition emissions, diesel, and secondary sulfate, respectively. (Figures from the supplemental material of Chen et al. (32)).

Although this study did not find associations between GAS and DIE sources and the rates of STEMI during the entire period (2014–2019), each 1.73 μg/m^3^ increase in PM_2.5_ concentration from the GAS source was associated with an increased rate of STEMI on lag day 0 in the 2014–2016 period (RR = 1.69; 95% CI = 0.98, 2.94), but not in the 2017–2019 period (RR = 0.79; 95% CI = 0.55, 1.13). This can perhaps be explained, in part, by our recent study (32). We found that although the monotonic trend in GAS over the period of 2010 to 2019 was positive and significant, the piecewise analysis found a breakpoint occurring around the middle of 2017 and a small downward trend to the end of 2019 suggesting changing GAS emissions during this period (Figure 1) (32). Alternatively, DIE remained constant according to the monotonic trend, but was decreasing slowly from the middle of 2012 according to the breakpoint analysis. Consistent with our study, Rich et al. reported an increased excess rate of hospitalizations for myocardial infarction (MI) associated with increased concentrations of PM_2.5_ from the GAS source on lag day 0 in New York State from 2005 and 2016 (2.3%; 95% CI = 0.1, 4.5%; IQR = 2.56 μg/m^3^), but not from the DIE source (0.4%; 95% CI = −0.5, 1.2%; IQR = 0.53 μg/m^3^) (25). They also found increased GAS concentrations associated with increased hospitalizations for ischemic stroke (excess rate = 3.5%; 95% CI = 1.0, 6.0%; IQR = 2.56 μg/m^3^) and an increase in DIE source (IQR = 0.53 μg/m^3^) associated with increased excess rates of congestive heart failure (0.7%; 95% CI = 0.2, 1.3%) and ischemic heart disease (0.6%; 95% CI = 0.1, 1.1%) hospitalizations on lag day 0. In addition, Sarnat et al. observed that cardiovascular emergency department (ED) visits were positively associated with same-day PM_2.5_ concentrations from mobile sources (RR range, 1.018–1.025) in Atlanta from 1998 to 2002 (40). In contrast, another Atlanta study (41) accounting for the uncertainty of source apportionment methods, reported no associations between mobile source PM_2.5_ (diesel- and gasoline-fueled vehicles) and ED visits for ischemic heart disease during 1998–2010. A possible mechanism for traffic PM_2.5_ sources associated with an increased risk of cardiovascular events is that traffic-related particles could contribute to both exogenous and endogenous reactive oxygen species (ROS).

Multiple prior studies have examined associations between organic carbon (OC) and adverse cardiovascular outcomes, with both positive (19, 42, 43) and negative (44–46) results reported. In addition, the long-term associations between SOC and cardiovascular events have been explored and their results were inconsistent (47, 48). However, current evidence about the short-term association is limited. Pennington et al. found each 1 μg/m^3^ increase in SOC concentrations was associated with ED visits for ischemic heart disease on lag day 0 (RR = 1.003; 95% CI = 0.997, 1.009) in Atlanta from 1998 to 2010 (41). Similarly, despite imprecision, our finding indicated a suggestive increased rate of STEMI associated with an increase in SOC concentration on lag day 0–6 (RR = 1.14; 95% CI = 0.89, 1.45; IQR = 0.42 μg/m^3^). The possible mechanism for the association between SOC and STEMI is likely related to the formation of secondary PM species including SOC, alongside the identification of increased PM_2.5_ toxicity, resulting in an increased risk of cardiovascular events (16, 49). SOC formation can result in concurrent oxidant species, and the related oxidative stress and inflammation could be potential drivers of adverse cardiovascular outcomes (22, 25). The imprecision in this study could be attributable to the limited sample size of the present study. Another possible factor to be considered is that the same pollutant values from one monitoring site were assigned for each subject on a specific day, regardless of how far they lived from the monitoring site, which likely cause underestimated effects.

Rather than single components being considered as causal, the combined effects of multiple constituents that possibly interact with environmental factors or combine with socioeconomic and biological characteristics may have substantial impacts on cardiac health (3). Secondary organic aerosol (SOA), derived from volatile organic compounds, is one such component. Fresh SOA is considered to contain peroxy radicals and peroxides and be strongly oxidizing (21, 22, 25, 50). Further, traffic PM sources may not only contribute to increased SOC concentrations (25, 26, 31, 49) but are also an important source of SOA and its precursors (3, 25, 51, 52).

A recent study (53) examined the association between SOA concentrations and cardiorespiratory disease mortality in the United States in 2016. It demonstrated that annual average SOA concentrations were strongly associated with county-level cardiorespiratory death rates, with a larger association per unit mass (*β* = 8.9 additional deaths per 100,000; 95% CI = 6.0, 12.0) than total PM_2.5_ (*β* = 1.4 additional deaths per 100,000; 95% CI = 0.5, 2.3). They considered that prior inconsistent results about associations of OC with adverse health outcomes could be, in part, explained by total OC lacking the distinction between primary organic aerosol (POA) and SOA (53). Although ambient PM_2.5_ concentrations are expected to decline in the future with strict air quality policies, SOA levels may relatively increase, resulting in increased health consequences per unit mass (49). Thus, future research should focus on the health effects of different PM components, including SOA and its constituents.

We did not observe increased rates of STEMI associated with SS, SN, RD, BB, OP, and RS. Similarly, Rich et al. reported no obvious associations of MI hospitalizations with SS, RD, BB, OP, and RS, while they found an association with SN (excess rate = 1.7%; 95% CI = 0.4, 3.0%; IQR = 1.53 μg/m^3^) on lag day 0–3 (25). Another study reported no clear associations between MI mortality and SS, SN, and BB (54). SS and SN, being unreactive particles, would not provide oxidative potential or the resulting reactive oxygen species and oxidative stress (25, 26). OP is thought to represent more aged SOA that has been transported into the area with little associated ROS (25, 55–57). As a result, the absence of OP association might reflect its low ROS concentration. Our null findings about RD and BB may be related to their heterogeneities. RD, representing non-exhaust traffic emissions, contains deposited soil and road surface material. Its effects on STEMI may differ by deposition rates, variability in reactivity, and environmental factors. Likewise, BB could have different PM compositional patterns due to various biofuels. RS does not generally include strongly oxidizing constituents.

Interestingly, although the difference in the association between SOC and the rate of STEMI between the two periods was not found, we observed that SS and GAS concentrations on lag day 0 associated with the rates of STEMI were different between the 2014–2016 and 2017–2019 periods, and their adverse effects on the triggering of STEMI might have a declining trend. Concentrations of sulfur-containing pollutants have been decreasing (58, 59) due to the implementation of air quality policies in New York State, such as particle traps required on heavy-duty diesel trucks and reductions in the sulfur content of diesel and home heating fuels. Also in Figure 1, SS shows a steady monotonic downward trend with only minor deviations showing the impacts of coal-fired power plant closures and reduced fuel sulfur concentrations. Consistent with our finding, Yount et al. (14) found that increased rates of STEMI were associated with increases in SO_2_ concentrations in previous 120 h (RR = 1.26; 95% CI = 1.03, 1.55; IQR = 0.59 ppb) during 2014–2016, but not in the 2017–2019 period (RR = 1.21; 95% CI = 0.87, 1.68). Our finding regarding GAS is also similar to our previous study (14), which showed an increased rate of STEMI associated with an increase in concentrations of black carbon, a marker of traffic pollution, in the previous hour (RR = 1.16; 95% CI = 0.99, 1.34; IQR = 0.30 μg/m^3^) in the 2014–2016 period instead of the 2017–2019 period (RR = 0.85; 95% CI = 0.72, 1.01). The reduction in the impact of GAS may be due to changes in PM components and/or PM mixtures resulting from air pollution regulations, including the introduction of Tier 3 light-duty vehicles. Inconsistent with our exception, we did not observe changes in the rate of STEMI associated with SOC after the Tier 3 regulation was implemented. It is estimated that only 36% of vehicles registered in New York State were Tier 3 in 2020 (14). Thus, the null finding of SOC is possibly attributed to the limited penetration of Tier 3 vehicles in the early Tier 3 implementation period (2017–2019). Consequently, the magnitude of the association between SOC and STEMI is not strong enough to be observed. In addition, when accounting for these results, some other possible reasons should be considered, such as healthcare improvements, public awareness and behavioral changes, and differences in study populations, as well as greater access to health care and medical insurance over the study period.

This study has several strengths, including the use of a well-defined STEMI study population treated at the Cath Lab in Rochester and the use of a case-crossover study design to control for non-time-varying factors and interactions between them, thereby reducing confounding by these factors. However, there are several limitations to be considered when interpreting our results. First, since PM_2.5_ sources and components were only measured every 3rd or 6th day, the number of subjects for whom exposure data were available largely decreased, thus reducing statistical power and precision. Second, all cases were assigned the same values of PM_2.5_ sources and components for a specific day from a single monitoring site without considering individual-specific differences, such as the distance from the emission source and/or the monitoring site, outdoor exposure duration, and protective measures. This assumption likely led to non-differential exposure misclassification and underestimated effects. Third, it is difficult for case-crossover designs combined with conditional logistic regression to fully adjust for possible overdispersion (60), which may cause larger confidence intervals than reported. Last, the 2014–2016 and 2017–2019 periods were selected based on the implementation timing of the new policy for Tier 3 light-duty vehicles. However, given that 2017–2019 is the early Tier 3 implementation period and the interventions generally phase in and take time to be effective, there are actually no well-defined time windows for the policies and emission changes. Therefore, the actual impact of this new policy should be further evaluated after the full implementation of Tier 3 through 2025.

## Conclusion

We used data on STEMI events treated at the University of Rochester Medical Center from 2014 to 2019, as well as the concentrations of PM_2.5_ sources and organic carbon measured in Rochester, NY. Inconsistent with our *a priori* hypothesis, increased rates of STEMI were not associated with increased GAS and DIE concentrations in the previous few days. However, in the 2014–2016 period, increased PM_2.5_ concentrations from the GAS source were associated with an increased rate of STEMI on the same day, which was not observed in the 2017–2019 period. Despite imprecision, our finding suggested that a short-term increase in SOC concentration might be associated with an increased rate of STEMI. We also found no association between rates of STEMI and POC or any other source-specific PM_2.5_ concentration. These negative results may be due to the potential changes in traffic emissions as well as the reduced statistical power and precision resulting from our limited sample size and potential exposure misclassification and effect underestimation. Furthermore, we observed significant differences in period-specific rate ratios for SS and GAS on the same day between the 2014–2016 and 2017–2019 periods, with a declining trend regarding adverse effects on the triggering of STEMI. This finding may be related to the changes in PM components and/or PM mixtures. Future work will be needed to further examine the effects of PM components and sources on triggering MI using a large sample after the Tier 3 light-duty vehicle policy is fully implemented in New York State.

## Data availability statement

The datasets presented in this article are not readily available because they contain protected health information and the authors are thus not permitted to release the data.

## Ethics statement

The studies involving humans were approved by University of Rochester Research Subjects Review Board. The studies were conducted in accordance with the local legislation and institutional requirements. The ethics committee/institutional review board waived the requirement of written informed consent for participation from the participants or the participants’ legal guardians/next of kin because the research involved no more than minimal risk to study subjects, the research could not be carried out without the waiver, and the waiver was judged not to adversely affect the rights and welfare of the subjects.

## Author contributions

TZ: Formal analysis, Writing – original draft. PH: Funding acquisition, Project administration, Conceptualization, Data curation, Formal analysis, Methodology, Supervision, Writing – review & editing. MU: Conceptualization, Funding acquisition, Writing – review & editing. DC: Funding acquisition, Conceptualization, Investigation, Writing – review & editing. ST: Funding acquisition, Conceptualization, Methodology, Writing – review & editing. SL: Funding acquisition, Project administration, Conceptualization, Methodology, Writing – review & editing. FL: Conceptualization, Data curation, Writing – review & editing. YC: Data curation, Formal analysis, Writing – review & editing. CY: Formal analysis, Writing – review & editing. DR: Conceptualization, Data curation, Formal analysis, Funding acquisition, Methodology, Project administration, Supervision, Writing – review & editing.

## Footnotes

^1^ www.epa.gov/aqs

^2^ https://www.ncdc.noaa.gov/cdo-web/datatools/lcd
